# Immunization Effects of a Novel α-Synuclein-Based Peptide Epitope Vaccine in Parkinson’s Disease-Associated Pathology

**DOI:** 10.3390/vaccines11121820

**Published:** 2023-12-05

**Authors:** Jun Sung Park, Riaz Ahmad, Kyonghwan Choe, Min Hwa Kang, Tae Ju Park, Myeong Ok Kim

**Affiliations:** 1 Division of Life Sciences and Applied Life Science (BK 21 Four), College of Natural Science, Gyeongsang National University, Jinju 52828, Republic of Korea; jsp@gnu.ac.kr (J.S.P.); riazk0499@gnu.ac.kr (R.A.); k.choe@gnu.ac.kr or kmh1020@gnu.ac.kr (M.H.K.); 2 Department of Psychiatry and Neuropsychology, School for Mental Health and Neuroscience (MHeNs), Maastricht University, 6229 ER Maastricht, The Netherlands; 3 Haemato-Oncology/Systems Medicine Group, Paul O’Gorman Leukaemia Research Centre, Institute of Cancer Sciences, College of Medical, Veterinary & Life Sciences (MVLS), University of Glasgow, Glasgow G12 0ZD, UK; t.park.1@research.gla.ac.uk; 4 Alz-Dementia Korea Co., Jinju 52828, Republic of Korea

**Keywords:** Parkinson’s disease (PD), α-synuclein, epitope, immunization, neuroinflammation

## Abstract

Parkinson’s disease (PD) is a chronic neurodegenerative disease that affects the central nervous system, specifically the motor system. It is mainly caused by the loss of dopamine due to the accumulation of α-synuclein (α-syn) protein in the striatum and substantia nigra pars compacta (SNpc). Previous studies have reported that immunization may be a potential preventive strategy for neurodegenerative diseases such as Alzheimer’s disease (AD) and amyotrophic lateral sclerosis (ALS). Therefore, the aim of the study was to design an α-syn specific epitope vaccine and investigate its effect in PD-related pathophysiology using an α-syn-induced mouse model. We used an in silico model to identify and design a non-toxic α-syn-based peptide epitope vaccine and, to overcome poor immunogenicity, the vaccine was coupled with immunogenic carrier proteins, i.e., ovalbumin (OVA) and keyhole limpet haemocyanin (KLH). Our results showed that vaccinated PD mouse models, especially with vaccines with carrier proteins, improved in motor functions compared with the non-vaccinated PD model. Additionally, the vaccinated groups showed increased immunoglobulin G (IgG) levels in the spleen and plasma as well as decreased interleukin-10 (IL-10) levels in the plasma. Furthermore, vaccinated groups, especially OVA and KLH groups, showed decrease in α-syn levels and increased dopamine-related markers, i.e., tyrosine hydroxylase (TH), vesicle monoamine transporter 2 (VMAT2), and dopamine transporter (DAT), and autophagy activities in the striatum and SNpc. Lastly, our data showed decreased neuroinflammation by reducing the activation of microglia and astrocytes and pro-inflammatory cytokines in the immunized groups, especially with OVA and KLH carrier proteins. Overall, these results suggest that vaccination, especially with immunogenic carrier proteins, is effective in reducing the accumulation of α-syn aggregates in the brain and ameliorate PD-related pathophysiology. Hence, further development of this approach might have a potential role in preventing the development of PD.

## 1. Introduction

Parkinson’s disease (PD) is the second most widespread age-related neurodegenerative disease, affecting over five million people throughout the world [1]. PD is characterized by dopaminergic neuronal loss in the substantia nigra (SN), the accumulation of α-synuclein (α-syn), chronic neuroinflammation, the overproduction of reactive oxygen species (ROS), and mitochondrial dysfunction [2,3]. The accumulation of α-syn, a neuronal unfolded protein, in the Lewy bodies and Lewy neurites has been known as the major pathological hallmark of PD [4,5]. Increased levels of α-syn have shown to reduce the levels of tyrosine hydroxylase (TH), an enzyme that converts tyrosine to dopamine [6,7,8]. Additionally, α-syn also regulates the synthesis and release of dopamine (DA) [9]. However, several studies showed that α-syn acts as a negative regulator of dopamine transporter (DAT) activity and is able to interact with and reduce the activity of DAT and vesicular monoamine transporter 2 (VMAT2), a transporter of monoamines such as serotonin and dopamine [10,11,12]. Furthermore, studies have shown that aggregated α-syn can activate glial cells, i.e., microglia and astrocytes, and lead to the cell death of dopaminergic neurons and the production of inflammatory cytokines [13,14,15,16,17].

Immunotherapy approaches targeting α-syn have been shown to amend α-syn pathology and functional deficits in PD mouse models and have advanced toward clinical development [18,19]. One of the most important functions of epitope design is to induce a strong and long-lasting immunoreactivity against the intended target which comes down to choosing the right epitope [20]. Raising epitope-specific antibodies is a primary mechanism of protection for many vaccines. For infectious diseases, often the targeted epitope, which binds to the antigen-binding region of IgG fragment, is a susceptibility site for neutralization by antibodies. Peptide vaccines utilize distinct peptide epitopes for B and T cells. The B-cell epitope refers to a particular fragment of the antigen/immunogen recognized by B-cell receptors on the B-cell surface, leading to the generation of antibodies during B-cell stimulation/maturation [20]. In addition, immunogenic carrier proteins such as ovalbumin (OVA) and keyhole limpet hemocyanin (KLH) have been investigated in neurodegenerative diseases such as Alzheimer’s disease (AD) [21,22]. Thus, targeting the formation and progression of α-syn opens a new and promising disease-modifying therapeutic strategy. For example, Schenk et al. first described vaccination strategy against amyloid-β in AD [23], while Masliah et al. reported the degradation of α-syn aggregates in PD mouse models after human α-syn immunization [24]. However, the study did not use a peptide-based epitope vaccine with immunogenic carrier proteins and did not investigate the PD pathophysiology in-depth. Additionally, there are some clinical trials that use passive and active immunization against α-synuclein for PD treatment [25].

Therefore, the aim of the current study was to examine whether passive immunization with an α-syn-based peptide epitope vaccine was able to recognize and clear α-syn aggregates and their related PD pathophysiology in a-syn-induced PD mouse models by identifying and designing a peptide epitope vaccine and investigating its biochemical and behavioral effects in animal models with Parkinson’s disease.

## 2. Materials and Methods

### 2.1. Animal and Peptide Epitope

Male wild-type C57BL/6N mice (25–30 g, 8 weeks old) were obtained from Samtako Bio (Osan, Republic of Korea). The mice were acclimatized for one week in the university animal house under a 12 h light/dark cycle at 23 °C, provided with food and water ad libitum. The protocols for the experimental procedures were evaluated and approved (Approval ID: GNU-200331-M0020) by the animal ethics committee (IACUC) of the Division of Applied Life Sciences, Department of Biology at Gyeongsang National University, Republic of Korea. Peptide epitope was custom-made based on the PEP1 sequence (VAEKTKEQVT), with acetylation (Ac) at the N terminal and a thiol group necessary for linking the carrier protein at the C terminal to attach the carrier protein (OVA and KLH) and combined by adding cysteine (C) amino acid (AC-VAEKTKEQVTC-OVA and Ac-VAEKTKEQVIC-KLH). The company Peptron (Daejeon, Republic of Korea) synthesized the peptide-based epitopes, both with and without carrier with a purity > 95%.

### 2.2. Immunizations

Mice (*n* = 10 per group) were immunized with a non-carrier protein (N) and two different carrier protein (OVA and KLH) vaccines. Using the synthesized α-synuclein antigen, in vivo experiments were performed as follows: α-syn antigen was administered by intraperitoneal (i.p.) injection, 15 μg/g of body weight once a week. A total of four injections were performed from six weeks to ten weeks of age, and after four weeks, an α-syn solution (2 μg/μL) dissolved in 1× phosphate-buffered saline (PBS) was injected into the ventricle. The α-synuclein pre-formed fibrils (PFF) preparation and injection was performed according to a published study [26]. Briefly, sonicated α synuclein pre-formed fibrils (StressMarq Biosciences Inc., Victoria, BC, Canada, Catalog No. SPR-322) were kept at room temperature during the duration of the surgical procedures. All mice were anesthetized with isoflurane and a pulled glass needle attached to a 10 μL Hamilton syringe was used to inject 4 μL of unilateral intrastriatal injections (2 μL each side; AP + 1.6, ML + 2.4, DV − 4.2; AP − 1.4, ML + 2.0, DV − 7.0 from the skull) of sonicated mouse α-syn PFF or DPBS at a rate of 0.5 μL/min. After each injection, the needles were not pulled out immediately but were kept in place for 1 min, retracted 0.5 mm, kept for another 2 min, and then were withdrawn slowly.

### 2.3. Open Field Test

Open field test was performed as described in our previous study [27]. Briefly, we used a white Plexiglas (40 × 40 cm in diameter, 40 cm in height) with its floor divided into 16 equally sized squares. Four squares were considered as the center and the other twelve squares along the walls as the periphery. In order to adjust to the new environment, the mouse was placed in the center of the box for few hours beforehand. Additionally, a calm and mutedly lit room was maintained in order to prevent distractions and sudden freezing behavior. Prior to each trail, the chamber was cleaned to remove any odor using ethanol solution (10%). The line crossing by the mouse and the time spent in the center were recorded by video tracking software 3.0 (Panlab, Holliston, MA, USA).

### 2.4. Rotarod Performance Test

Rotarod performance test was conducted as described in our previous study [28]. Briefly, before the formal trial, each mouse was given a three-day pre-training session. During the formal testing, each mouse was placed on the rotarod with a rolling speed of 12 or 20 rpm. The time for which the mouse stayed on the rotarod and the latency to fall were observed and measured.

### 2.5. Protein Extraction from Mouse Brain

After the behavioral analyses, the mice were anesthetized with ketamine/xylazine and then sacrificed in accordance to our previous study [29]. Briefly, the brain was quickly removed and the striatum and SNpc were separated carefully. The sections were homogenized using PRO-PREPTM extraction solution (iNtRON Biotechnology, Burlington, NJ, USA), centrifuged at 13,000 rpm at 4 °C, and stored at −80 °C until needed.

### 2.6. Western Blot Analysis

Western blot analysis was conducted according to our previous studies [30,31]. Briefly, in order to load equal amounts of protein samples, Bradford assay (Bio-Rad protein assay kit, Bio-Rad Laboratories, Hercules, CA, USA) was used to measure protein concentration. Next, the samples and protein marker (GangNam-STAIN, iNtRON Biotechnology, CA, USA) were electrophoresed on SDS-PAGE, then transferred to polyvinylidene difluoride membranes (PVDF) (Millipore, Burlington, MA, USA). The membrane was blocked in 5% skim milk, and then was incubated in primary antibody overnight at 4 °C (Table 1). On the next day, the membrane was washed with 1 × PBS with Tween 20 (PBST), blocked with horseradish-peroxidase-conjugated secondary antibody for 1 h, washed with 1 × PBST, then detected using an ECL detection reagent (EzWestLumiOne, ATTO, Tokyo, Japan) according to the manufacturer’s instructions. Lastly, the immunoblot bands were detected using an X-ray film. The films were scanned, measured using ImageJ software version 1.53k, and presented graphically.

### 2.7. Immunofluorescence Staining

Immunofluorescence staining was conducted as previously described in our studies [32,33]. Briefly, the brain was fixed in 4% paraformaldehyde for 24 h, and then it was dehydrated in 20% sucrose solution. Next, the brain sections, i.e., spleen, SNpc, and striatum, were cryosectioned at 20 μm using a cryostat (CM1860, Leica Biosystems, Wetzlar, Germany). The slides were washed twice in filtered PBS, treated with proteinase K, blocked with 5% goat serum, and then primary antibodies were applied overnight at 4 °C (Table 1). Next day, the slides were washed twice, treated with fluorescein isothiocyanate (FITC)-labeled secondary antibodies, washed, and then treated with 4′,6-diamidino-2-phenylindole (DAPI). Lastly, the slides were covered using coverslips with a fluorescent mounting medium and, using a confocal scanning microscope (FV1000MPE, Olympus, Shinjuku City, Tokyo, Japan), the images were captured and were quantified via the relative integrated densities using ImageJ software (version 1.53k).

### 2.8. ELISA

Enzyme-linked immunosorbent assay (ELISA) kit (MBS704754, MBS2708011, MyBioSource, CA, USA) was used to quantify IL-10 and IgG1 concentrations in mouse serum, following the manufacture’s protocol. Briefly, mouse serum was incubated for 2 h at 37 °C per well in a 96 well plate. Then, the serum was removed and incubated with Biotin-antibody for 1 h at 37 °C. Next, the plate was washed 3 times, HRP-avidin was added, it was covered with adhesive strip, and incubated for 1 h at 37 °C. Afterwards, TMB Substrate was added, and then Stop Solution was added. Lastly, the optical density of each well was measured using a microplate reader set at 450 nm.

### 2.9. Statistical Analysis

All data are presented as mean ± standard error of the mean (SEM). Prior to any statistical analysis, the Shapiro–Wilk test for normality was performed. Group comparisons were performed by one-way analysis of variance (ANOVA) followed by a Bonferroni multiple comparison test. *p*-values less than 0.05 were considered statistically significant. All statistical analyses and graphs were generated using GraphPad Prism (version 8.0.2).

## 3. Results

### 3.1. Identifying and Designing α-Synuclein Based Peptide Epitope Vaccine

**Figure 1:**
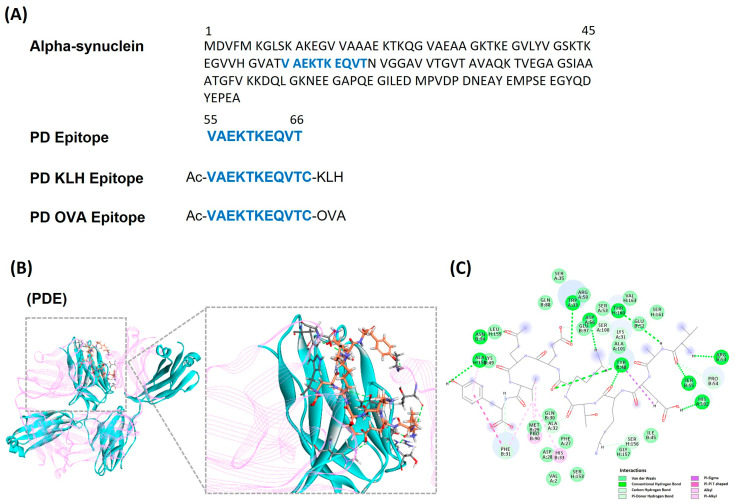
In silico model to identify and design the α-syn-based peptide epitope vaccine. (**A**) Identifying the 10 peptide-based (VAEKTKEQVT) to be used to design the peptide epitope vaccine. Three different vaccines were designed: without immunogenic carrier protein (PD Epitope), and with immunogenic carrier proteins, i.e., OVA (PD OVA Epitope) and KLH (PD KLH Epitope). (**B**,**C**) Interaction residue with B cell through structure of α-syn antigen peptide and docking simulation. See Supplementary Figures for further details.

Based on the α-syn sequence input (Figure 1A), the B-cell epitope prediction tool in the Immune Epitope Database (IDEB) identified several predicted peptides (Supplementary Figure S1A–C). Next, by calculating the binding effectiveness and stability using a molecular docking simulation via the genetic optimization for ligand docking (GOLD), three peptides (PEP1, VAEKTKEQVT; PEP2, AEKTKEQVTN; PEP3, EKTKEQVTNV) were selected. These peptide candidates underwent ten GOLD attempts, of which, PEP1 had the highest average score (Supplementary Figure S2A). Then, using the Toxinpred tool, PEP1 was predicted to be non-toxic, and was thus selected as the α-syn specific epitope (PDE-N; Figure 1A; Supplementary Figure S2B). Furthermore, peptide modification was required to induce B-cell activity. Therefore, a carrier protein, KLH (PDE-KLH) and OVA (PDE-OVA), was attached to PEP1 in the C terminal through Thiol group modification, via a cysteine side chain. In addition, to create a peptide antigen closer to the actual α-synuclein, acetylation (Ac) was attached at the N terminal. This modification allowed for the stability of the peptide against various degrading enzymes and allowed higher immunoreactivity (Figure 1A). Lastly, through docking simulations between PEP1 and the B cells, the simulation showed that the residues of B cells bind to PEP1 (Figure 1B,C; Supplementary Figure S2C). Therefore, based on these in silico findings, PEP1 was selected as the ideal α-syn-based epitope vaccine.

### 3.2. Immunization Epitope Vaccine Abrogated Motor Dysfunction in α-syn Induced PD Mouse Model

**Figure 2:**
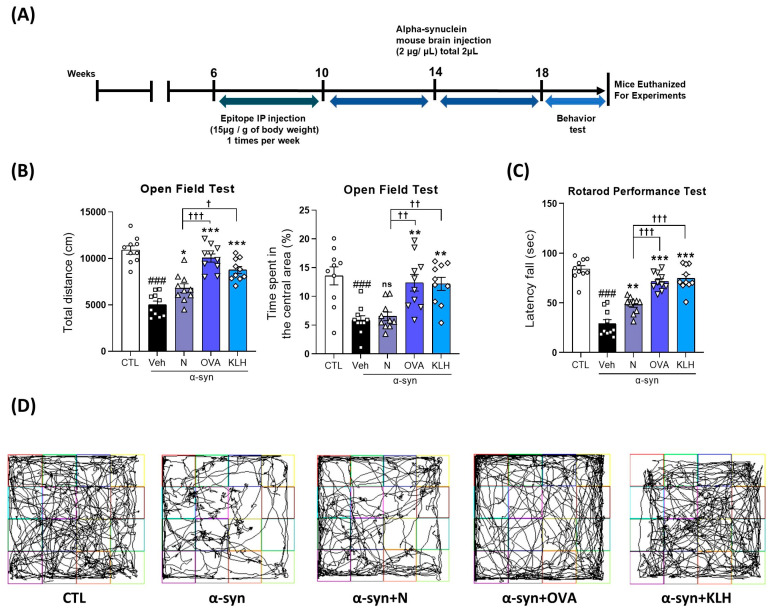
Epitope treatment improved motor dysfunction in α-syn-induced PD model. (**A**) Study design of the study. (**B**) Quantitative analysis of the total distance (left panel) and time spent in the central area covered (right panel) by the mice in the open field box (*n* = 10 per group). (**C**) Quantitative analysis of the latency to fall by the mice in the rotarod performance test (*n* = 10 per group). (**D**) Representative images of the open field box. Comparisons: ^#^ control (CTL) with saline-treated (Veh) α-syn-induced PD model; * Veh group with epitope-treated group [non-carrier protein (N) and carrier-protein (OVA and KLH); ^†^ Non-carrier protein (N) with carrier protein (OVA and KLH). Data are presented as mean ± SEM. *^/†^ *p* < 0.05, **^/††^ *p* ≤ 0.01, ^###/^***^/†††^ *p* ≤ 0.001, and non-significant (ns).

As mentioned previously, the main symptom of PD is motor dysfunction and the accumulation of α-syn has been reported to be a key player. Therefore, we examined motor dysfunction by conducting an open field test (OFT) and rotarod performance test (Figure 2A). Our OFT demonstrated that the α-syn-treated mice significantly reduced the total distance in the open field box compared with the control group. However, in comparison with the α-syn mice group, the mice with epitope treatment (N, OVA, KLH) showed significant improvements in total distance, for all the epitope groups, and time spent in the central area, only with the carrier protein groups (Figure 2B,D). Additionally, in comparison within the treatment groups, epitopes with a carrier (OVA and KLH) were significantly better than non-carrier attached epitopes (Figure 2B,D). In the rotarod test, we report only the latency to fall. Thus, the latency to fall from the accelerating rod was observed among all five groups of mice. The α-syn-treated mice were observed to remain for a short time on the rod, while the mice vaccinated with (non)carrier groups improved their stability and stayed significantly longer on the accelerated rod, and the carrier groups showed higher latency fall times than the non-carrier group (Figure 2C). Overall, our data show that the vaccine groups showed significant improvement in motor functions compared with the non-vaccinated group and vaccines with either OVA or KLH carrier proteins exhibited better improvement than the non-carrier group.

### 3.3. Changes in IgG in the Spleen and Plasma after the Administration Epitope Vaccines in α-syn-Induced PD Mouse Model

**Figure 3:**
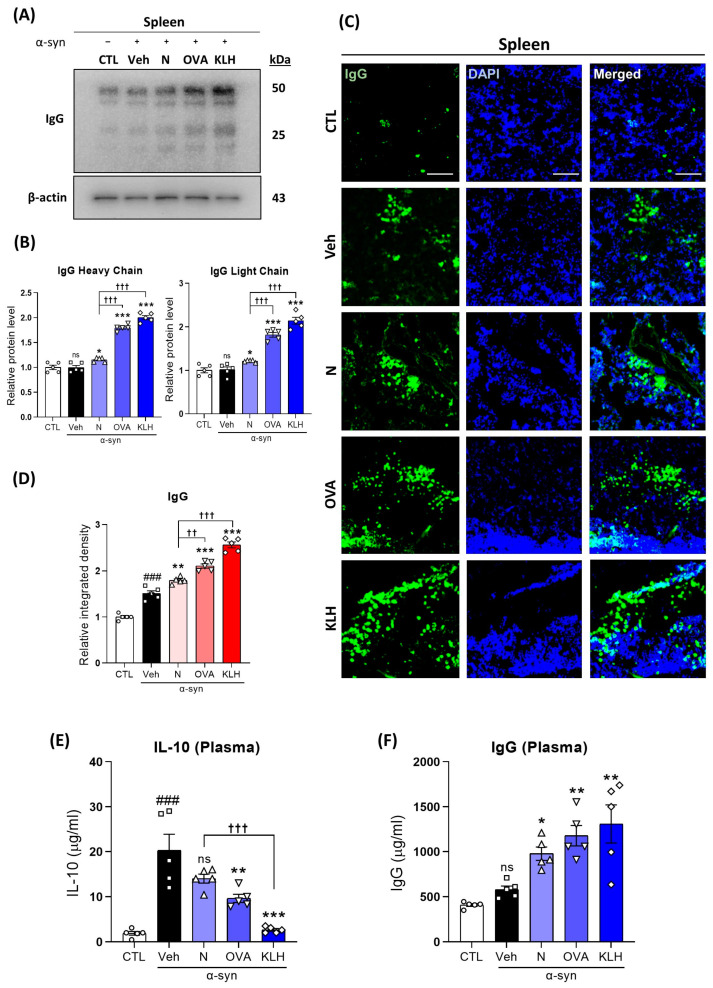
Epitope treatment initiated an immune response. (**A**,**B**) Western blot analysis measuring the expression of IgG (heavy chain [50 kDa] and light chain [25 kDa]) in the spleen (*n* = 5 per group). (**C**,**D**) Immunofluorescence staining measuring the expression of IgG reactivity (green) co-stained with DAPI (blue) in the spleen (*n* = 5 per group). (**E**,**F**) Quantitative measurement of (**E**) IL-10 and (**F**) IgG levels in the plasma (*n* = 5 per group). Scale bar present 100 μm. Comparisons: ^#^ control (CTL) with saline-treated (Veh) α-syn-induced PD model; * Veh group with epitope treated group [non-carrier protein (N) and carrier-protein (OVA and KLH); ^†^ Non-carrier protein (N) with carrier protein (OVA and KLH). Data are presented as mean ± SEM. * *p* < 0.05, **^/††^ *p* ≤ 0.01, ^###/^***^/†††^ *p* ≤ 0.001, and non-significant (ns).

Next, we investigated the IgG levels in the spleen and in the plasma of all five groups of mice to examine whether the vaccines caused immunoreactivity. Our results showed that IgG levels were increased in the mice vaccinated with (non)carriers compared with the control and vehicle treatment groups (Figure 3A,B) and OVA and KLH vaccines showed significantly higher IgG levels compared with the non-carrier group. This observation was further confirmed by immunofluorescence analysis and partially in the plasma IgG level, in which vaccines showed higher plasma IgG levels, but no differences were shown between the vaccine variations (Figure 3C,D,F). Furthermore, plasma IL-10 levels were significantly lower in mice that received the carrier protein vaccines compared with α-syn mice (Figure 3E). Therefore, our data confirmed that the selected antigen binds to B cells to produce α synuclein antibodies.

### 3.4. Immunization with Epitope Vaccine Eliminated α-syn Protein Aggregation and Increased Autophagy in α-syn-Induced PD Mouse Model

**Figure 4:**
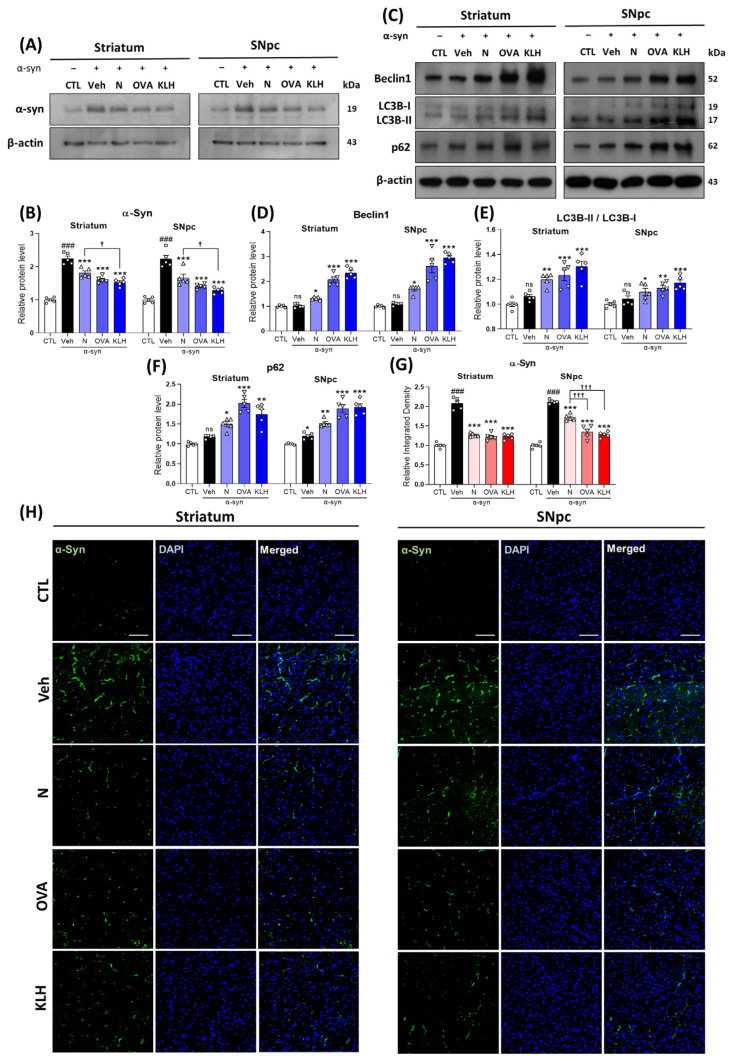
Epitope treatment reversed the α-syn and autophagy levels in the brain. (**A**,**B**) Western blot analysis showing the expression of α-syn in the striatum and SNpc (*n* = 5 per group). (**C**–**F**) Western blot analysis showing the expression of autophagy-related markers, i.e., (**D**) beclin-1, (**E**) LC3B-II/I, and (**F**) p62 in the striatum and SNpc (*n* = 5 per group). (**G**,**H**) Immunofluorescence staining measuring the expression of α-syn (green) co-stained with DAPI (blue) in the striatum and SNpc (*n* = 5 per group). Scale bar present 50 μm. Comparisons: ^#^ control (CTL) with saline-treated (Veh) α-syn-induced PD model; * Veh group with epitope treated group [non-carrier protein (N) and carrier-protein (OVA and KLH); ^†^ Non-carrier protein (N) with carrier protein (OVA and KLH). Data are presented as mean ± SEM. *^/†^ *p* < 0.05, ** *p* ≤ 0.01, ^###/^***^/†††^ *p* ≤ 0.001, and non-significant (ns).

Our data demonstrated that all three groups of epitope vaccine induced effective immune responses and reduced the expression of α-syn in the striatum and SNpc compared with the non-vaccinated group. Additionally, between the vaccines, the vaccine with the OVA carrier did not show statistical difference between the non-carrier vaccines, while on the other hand, the vaccine with the KLH carrier showed a significant reduction compared with the non-carrier protein vaccine (Figure 4A,B). To validate our findings, we further examined this expression using immunofluorescence analysis. Similarly to our previous findings, vaccinated mice showed significant reductions in α-syn compared with the non-vaccinated group, but both vaccines with carrier proteins showed significant improvement in α-syn reduction in the SNpc, while this was not shown in the striatum (Figure 4G,H). Moreover, our data showed increased autophagy function, in which beclin 1, LC3B-II/I ratio, and p62 levels were significantly higher in the vaccinated groups, especially in the OVA and KLH groups (Figure 4C–F).

### 3.5. Immunization with Epitope Vaccine Reversed Dopamine-Related Markers in α-syn-Induced PD Mouse Model

**Figure 5:**
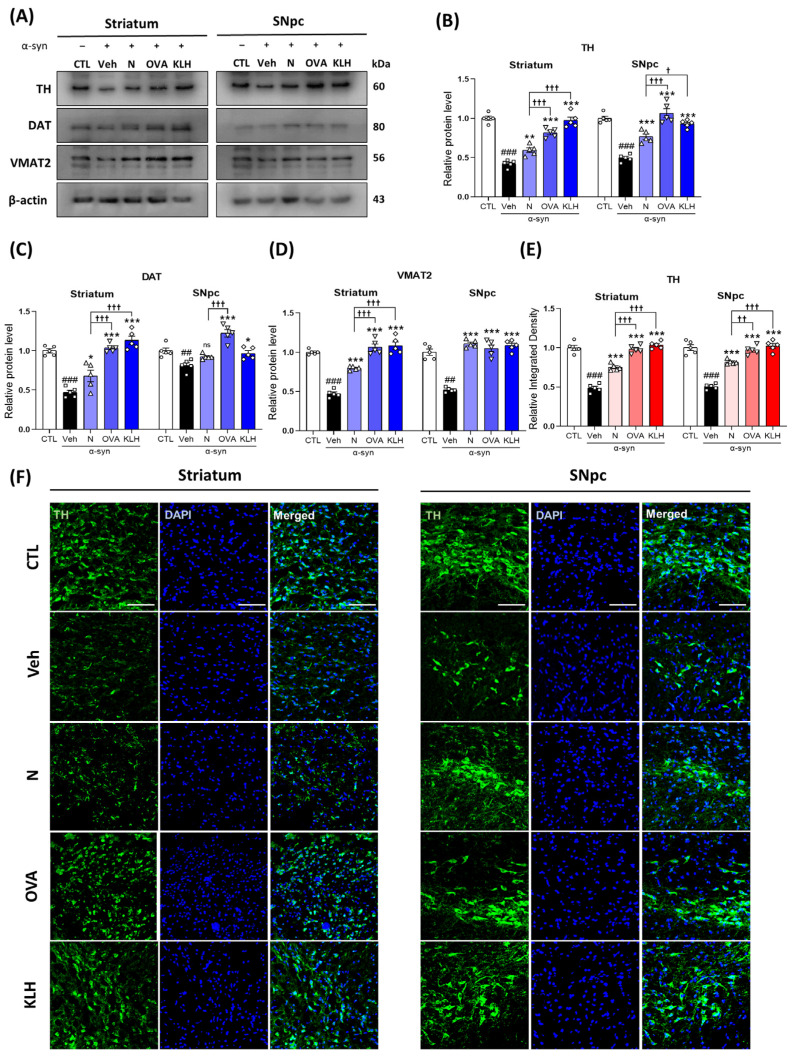
Epitope treatment increased dopamine-related markers in the brain. (**A**–**D**) Western blot analysis measuring the expressions of (**B**) tyrosine hydroxylase (TH), (**C**) dopamine transporter (DAT), and (**D**) vesicular monoamine transporter 2 (VMAT2) in the striatum and SNpc (*n* = 5 per group). (**E**,**F**) Immunofluorescence staining measuring the expression of TH (green) co-stained with DAPI (blue) in the striatum and SNpc (*n* = 5 per group). Scale bar present 50 μm. Comparisons: ^#^ control (CTL) with saline-treated (Veh) α-syn-induced PD model; * Veh group with epitope-treated group [non-carrier protein (N) and carrier-protein (OVA and KLH); ^†^ Non-carrier protein (N) with carrier protein (OVA and KLH). Data are presented as mean ± SEM. *^/†^ *p* < 0.05, **^/††^ *p* ≤ 0.01, ^###/^***^/†††^ *p* ≤ 0.001, and non-significant (ns).

As mentioned previously, decreases in dopamine are a leading cause for developing PD symptoms and the accumulation of α-syn is a key player in dopamine loss. Therefore, to examine the potential efficacy of the anti α-syn antibodies generated by epitope vaccines, we investigated the protein expression of key dopamine-related markers, i.e., TH, DAT, and VMAT2 in the striatum and SNpc. Our data showed that vaccinated mice groups significantly increased TH, DAT, and VMAT2 levels compared with the non-vaccinated group and vaccines with carrier proteins showed further enhanced TH, DAT, and VMAT2 levels than the non-carrier vaccine (Figure 5A–D). Similar findings were observed in the immunofluorescence TH staining, in which the TH signals were reduced in the α-syn-treated group as compared with the control, and the vaccinated groups exhibited significant increases, of which, the vaccine with carrier proteins showed higher TH levels than the non-carrier protein vaccine (Figure 5E,F; Supplementary Figure S3). Additionally, our data showed that TH was decreased and α-syn was increased in the ventral tegmental area (VTA) in the PD model, while our vaccine groups showed the opposite (Supplementary Figure S4). Lastly, we stained for neuronal nuclei (NeuN) to check the cell viability and our results showed that our vaccines increased the number for NeuN-stained cells in both the striatum and the SNpc and the protein expression of postsynaptic density protein 95 (PSD-95) was decreased in the PD model, while it was increased in the vaccinated model (Supplementary Figure S5).

### 3.6. Epitope Vaccine Immunization Decreases Glial Cell Activation in α-syn-Induced PD Mouse Model

**Figure 6:**
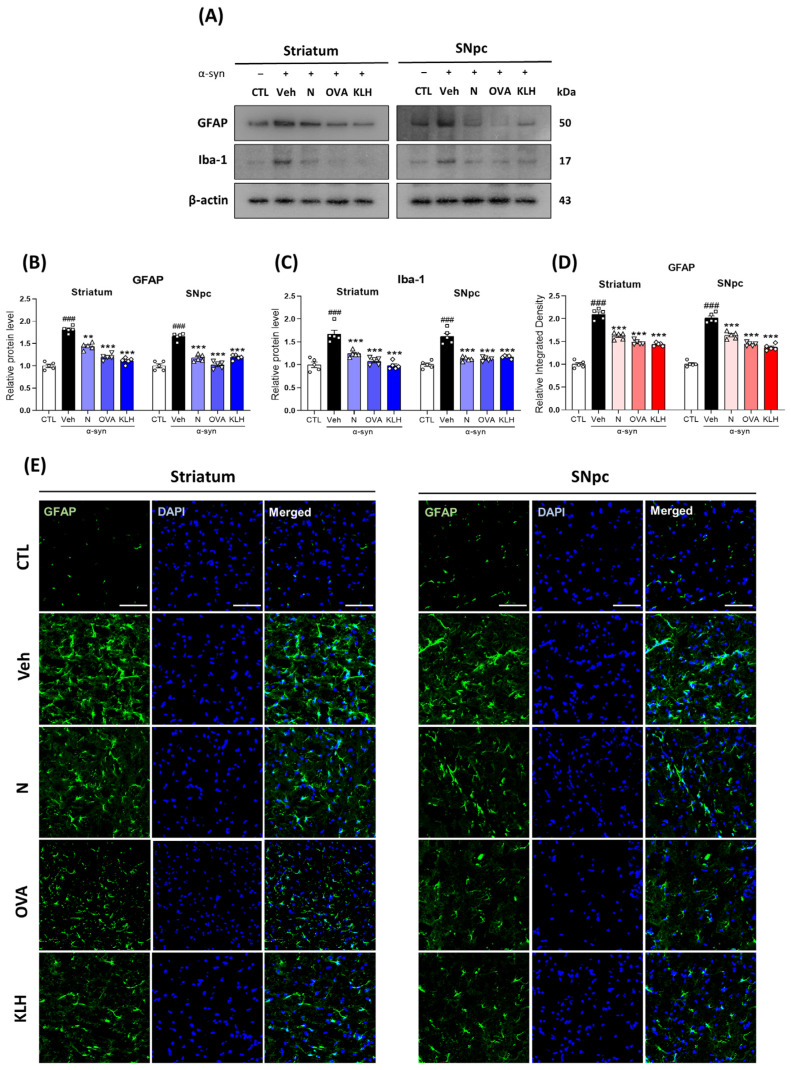
Epitope treatment reversed the glial cell activation in the brain. (**A**–**C**) Western blot analysis of (**B**) glial fibrillary acidic protein (GFAP) and (**C**) allograft inflammatory factor 1 (Iba-1) in the striatum and SNpc of mice (*n* = 5 per group); (**D**,**E**) Immunofluorescence staining measuring the expression of GFAP (green) co-stained with DAPI (blue) in the striatum and SNpc (*n* = 5 per group). Scale bar present 50 μm. Comparisons: ^#^ control (CTL) with saline-treated (Veh) α-syn-induced PD model; * Veh group with epitope-treated group [non-carrier protein (N) and carrier-protein (OVA and KLH). Data are presented as mean ± SEM. ** *p* ≤ 0.01, ^###/^*** *p* ≤ 0.001, and non-significant (ns).

To determine whether peptide-based epitope vaccines could reverse α-syn-induced activation of astrocytes and microglia in the striatum and SNpc of mouse brain, we investigated glial-related markers, i.e., GFAP and Iba-1. Our results indicated a significant increase in both GFAP and Iba-1 expressions in the α-syn-induced group compared with the control mice. However, the expressions were significantly reduced in vaccinated groups (Figure 6A–C). Additionally, vaccines with carrier proteins significantly decreased GFAP and Iba-1 levels more than non-carrier vaccines in the striatum, but only the OVA carrier showed significant differences in the SNpc (Figure 6A–C). To confirm this observation, we also investigated GFAP expression using immunofluorescence staining. Similar to our western blot analysis, the staining also showed that GFAP was significantly increased in the α-syn group compared with the control mice. Furthermore, the vaccinated groups showed lower GFAP levels and, in both striatum and SNpc, vaccines with carrier proteins exhibited a further decrease in expression than the non-carrier proteins (Figure 6D,E).

### 3.7. Epitope Vaccine Immunization Reduced the Expression of Inflammatory Cytokines in α-syn-Induced PD Mouse Model

**Figure 7:**
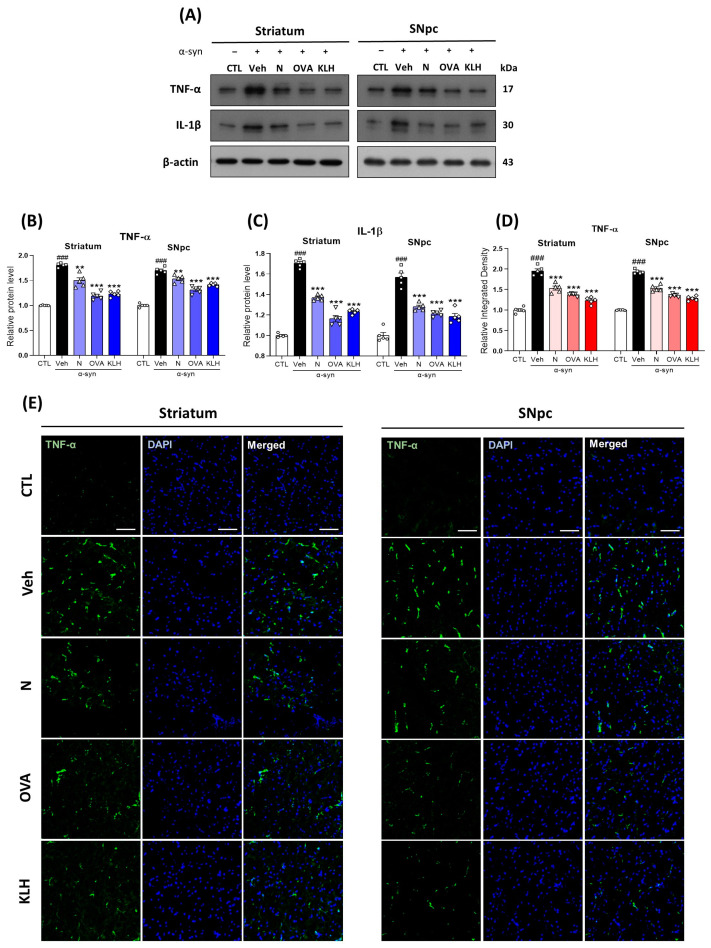
Epitope treatment alleviates the expression of inflammatory cytokines in the brain. (**A**–**C**) Western blot analysis showing the expression of (**B**) tumor necrosis factor-α (TNF-α) and (**C**) interleukin-1β (IL-1β) in the striatum and SNpc (*n* = 5 per group). (**D**,**E**) Immunofluorescence staining measuring the expression of TNF-α (green) stained with DAPI (blue) in the striatum and SNpc (*n* = 5 per group). Scale bar present 50 μm. Comparisons: ^#^ control (CTL) with saline-treated (Veh) α-syn-induced PD model; * Veh group with epitope-treated group [non-carrier protein (N) and carrier-protein (OVA and KLH). Data are presented as mean ± SEM. ** *p* ≤ 0.01, ^###/^*** *p* ≤ 0.001, and non-significant (ns).

Since glial cells are associated with neuroinflammation, we examined the effects of peptide-based epitope vaccines on α-syn-mediated inflammatory cytokines. Our immunoblot results showed that, compared with the control group of mice, the α-syn group showed increased protein expression levels of TNF-α and IL-1β. However, the vaccinated groups, especially OVA and KHL groups, exhibited significant reductions in TNF-α and IL-1β levels (Figure 7A–C). We validated our findings in the immunofluorescence analysis, as it also suggested that TNF-α immunoreactivity significantly increased in the α-syn group as compared with the control mice group. However, immunization significantly reduced the immunofluorescence reactivity of TNF-α in both the striatum and SNpc regions (Figure 7D,E). Lastly, our co-staining of TNF-α with NeuN showed that TNF-α expression is co-localized in the neurons (Supplementary Figure S6).

## 4. Discussion

The aim of the current study was to design an α-syn-based peptide epitope vaccine and study its effect on PD pathophysiology using an α-syn-induced PD mouse model. Our study designed three different types of vaccines, with (OVA and KLH) and without (N) immunogenic carrier proteins. Based on our data, the vaccines were safe and effective as mice treated with the vaccines showed improvement in motor function and PD-related pathology markers such as α-syn, dopamine, and neuroinflammation in the striatum and SNpc. Additionally, vaccines with carrier proteins showed enhanced immunogenicity which resulted in better treatment than the vaccine without a carrier protein.

α-synuclein is mainly expressed in the presynaptic terminals of neurons and, in normal conditions, naturally exists as unfolded monomers. It helps regulate the release of dopamine, which is important in controlling voluntary and involuntary movements. However, when the concentration of α-synuclein in the brain increases, it forms a beta-sheet structure and forms abnormal α-synuclein aggregates [34]. The residues essential for misfolding are sequence 71–82, and this region has been shown to be capable of aggregation alone [35]. In the case of our peptide, it was different from the 71–82 sequence that can aggregate alone, and our peptide was too short to form a beta-sheet structure for aggregation. Additionally, the α-synuclein PFF model offers insights into both PD pathophysiology and the effectiveness of therapeutic approaches in a relevant in vivo model. This includes investigating the mechanisms that trigger Lewy body formation and proliferation and how our epitope prevents these mechanisms. Furthermore, it has advantages for screening and efficacy verification. In addition, the PFF model presents some advantages over previous disease models. For example, while other models overexpress human WT or mutant α-syn, a small amount of non-phosphorylated PFFs can transform endogenously expressed α-syn into aggregates, resulting in a more physiologic impact of α-syn aggregates than in viral vector-based and transgenic models [36]. Additionally, the PFF model has considerable temporal and spatial resolution since the development over time of PFF-induced degeneration is similar to human conditions, in which early α-syn pathophysiology symptoms such as dopamine dysfunction leading to motor symptoms can be observed in the PFF models. Thus, this model allows us to follow the progression and development of α-syn aggregates from early formation to neuronal death [36,37]. Besides the PFF model, other mouse models, such as the 1-methyl-4-phenyl-1,2,3,6-tetrahydropyridine (MPTP)-induced PD model, have shown that the therapeutic immunization of Cop-1 and OVA protected dopaminergic neurons [38].

To raise antibodies against small non-immunogenic molecules, it is often necessary to couple antigens to carrier proteins such as OVA and KLH. KLH is used as an immunogenic neo-antigen for vaccine development since it is a clinical grade product that has low-grade toxicity and potent immunogenicity [39]. Additionally, OVA can induce humoral and cellular immune responses [40]. Previous studies showed the strong immune response of OVA conjugated with poly-GA and KLH conjugated with Aβ_3–10_, which improved cognitive function, rescued motor deficits, and ameliorated mitochondrial dysfunction in a C9orf72 mouse model of amyotrophic lateral sclerosis (ALS) and a Tg-APPswe/PSEN1dE9 mouse model of AD, respectively [21,22]. Moreover, IgG is the most common type of antibody found in the human blood and it is released by plasma B cells [41]. Additionally, spleen is a key lymphoid organ for B-cell development [42] and because our data have shown that the vaccines, especially with carrier proteins, significantly increased the IgG levels in both spleen and plasma, this indicates that the vaccines caused an immunogenicity.

The main symptom of PD is motor dysfunction due the accumulation of α-syn and the decrease in dopaminergic neurons in the striatum and SNpc brain regions [43]. Our α-syn-specific epitopes, especially with carrier proteins, induced a strong anti-α-syn response and significantly reduced the expression of α-syn. This was consistent with a study that showed that immunotherapy reduced the abnormal accumulation of α-syn in the neuronal cell bodies and synapses and ameliorated the loss of synaptophysin-immunoreactive nerve terminals in hα-syn Tg mice [24]. Furthermore, increases in α-syn have led to alterations in the autophagy lysosomal pathway [44]. The peptide-based epitope in this study binds to B cells and produce antibodies that bind to α-synuclein. When B cells produce antibodies, they expose the antigen to T cells to attract additional immune system components. Exposed epitope antigens induce autophagy in antigen-presenting cells (APCs), which induce CD4 and CD8 T-cell-dependent Th1 immunity. As a result, they reduce CD4 T-cell responses, thereby increasing the production of antibodies against α-syn [45]. Therefore, autophagy-related markers were investigated to evaluate the effects of a peptide-based epitope vaccine. Studies have shown that the overexpression of beclin-1, which results in decreased cell death and increased autophagy activity, was linked with reduced α-syn accumulation. Moreover, the same study also reported that the overexpression of beclin-1 elevated LC3 levels [46]. Furthermore, another study showed that the inactivation of LC3B enhances α-syn accumulation, while activation inhibited clumping and facilitated α-syn degradation [47].

Additionally, higher levels of α-syn have also been found to affect dopamine and its related markers, such as to reduce active TH, an enzyme involved in the production of dopamine, VMAT2, transporters for biogenic monoamines such as serotonin and dopamine from the cytoplasm into the vesicles, and DAT, a presynaptic dopamine transporter, activities [27]. Increased cytosol concentrations of dopamine due to the reduction in VMAT2 activity by α-syn have been proposed as a possible neurotoxic pathway in PD [48] and DAT levels decreasing by reducing the level of TH [49]. Since our α-syn-specific epitopes have shown to decrease α-syn accumulation in the striatum, SNpc, and VTA, this may explain the increased levels of TH, VMAT2, and DAT as well. Moreover, dopamine is a crucial for motor function [43], thus, through the positive effects of the epitopes, this may be the reason for the improved motor controls such as general activity level, motor coordination, and balance in our study.

Lastly, several studies have also indicated that misfolded α-syn may trigger microglial activation [50,51]. Recent work has even suggested that peptides derived from a-syn can trigger an autoimmune component to PD, thus α-syn and neuroinflammation mediated by the inflammatory response through microglial activation have been suggested to potentiate each other [50,52]. Moreover, microglial activation promotes further α-syn pathology by increased nitric oxide (NO) production, which in turn can induce nitration of α-syn in neighboring neurons and result in cell death [53]. Consistent with these results, our results also suggest that vaccination, especially with carrier proteins, can reduce the activation of microglia and astrocytes, which may occur because of the decline of α-syn aggregation. Additionally, the accumulation of α-syn in glial cells has been associated with an increase in the expression of pro-inflammatory cytokines [54]. Misfolded α-syn may activate microglia through the increased expression of TNF-α and IL-1β [55,56]. These cytokines have been reported to induce the death of dopaminergic cells and to cause neurodegenerative disease [57].

## 5. Conclusions

Overall, our findings support that our α-syn-specific vaccine showed an immunoreactivity against α-syn, hence reducing PD pathophysiology in α-syn-induced mouse models. Furthermore, vaccines with immunogenic carrier proteins, i.e., OVA and KLA, further ameliorate PD pathophysiology compared with a non-carrier protein vaccine. Therefore, these findings provide insights into the potential prevention strategy to protect against PD.

## Figures and Tables

**Table 1:** List of antibodies.

Antibody	Host	Application	Manufacturer	Catalog #	Concentration
α-syn	Mouse	WB/IF	Santa Cruz Biotechnology, Dallas, TX, USA	SC58480	1:1000/1:100
TH	Mouse/Rabbit	WB/IF	Santa Cruz Biotechnology Cell Signaling, Danvers, MA, USA	SC25269 E2L6M	1:1000/1:100
DAT	Rat	WB	Santa Cruz Biotechnology	SC32259	1:1000
VMAT2	Mouse	WB	Santa Cruz Biotechnology	SC374079	1:1000
IgG	Mouse	WB/IF/ELISA	Santa Cruz Biotechnology MyBioSource, San Diego, CA, USA	SC515946 MBS2708011	1:1000/1:100
IL-10	Mouse	ELISA	MyBioSource	MBS704754	1:100
GFAP	Mouse	WB/IF	Santa Cruz Biotechnology	SC33673	1:1000/1:100
Iba-1	Mouse	WB	Santa Cruz Biotechnology	SC398406	1:1000
TNF-α	Mouse	WB/IF	Santa Cruz Biotechnology	SC52746	1:1000/1:100
IL-1β	Mouse	WB	Santa Cruz Biotechnology	SC32294	1:1000
Beclin1	Mouse	WB	Santa Cruz Biotechnology	SC48341	1:1000
LC3B	Rabbit	WB	Abcam, Cambridge, UK	AB48394	1:1000
p62	Mouse	WB	Santa Cruz Biotechnology	SC48402	1:1000
PSD-95	Mouse	WB	Santa Cruz Biotechnology	SC71933	1:1000
NeuN	Rabbit	IF	Cell Signaling	D4G4O	1:100

Abbreviation: α-syn, α-synuclein; TH, tyrosine hydroxylase; DAT, dopamine transporter; VMAT2, vesicular monoamine transporter 2; IgG, immunoglobulin G; IL-10, interleukin-10; GFAP, glial fibrillary acidic protein; Iba-1, allograft inflammatory factor 1; TNF-α, tumor necrosis factor-α; IL-1β, interleukin-1β; PSD-95, postsynaptic density protein 95; WB, western blot; IF, immunofluorescence; ELISA, enzyme-linked immunosorbent assay.

## Data Availability

Data are contained within the article.

## Supplementary Materials

The following supporting information can be downloaded at: https://www.mdpi.com/article/10.3390/vaccines11121820/s1, Figure S1: Algorithms for predicting B cell epitope of α-syn using the Immune Epitope Database (IDEB); Figure S2: Examination of the three candidate peptides; Figure S3: Immunofluorescence double staining of α-synuclein (α-syn) and tyrosine hydroxylase (TH) in the striatum and the substantia nigra pars compacta (SNpc); Figure S4: Immunofluorescence double staining of α-synuclein (α-syn) and tyrosine hydroxylase (TH) in the ventral tegmental area (VTA); Figure S5: Peptide-based epitope vaccines increased neurons in the striatum and substantia nigra pars compacta (SNpc); Figure S6: Immunofluorescence double staining of tumor necrosis factor α (TNFα) and neuronal nuclei (NeuN) in the striatum and substantia nigra pars compacta (SNpc).
