# Correction: Park et al. Immunization Effects of a Novel α-Synuclein-Based Peptide Epitope Vaccine in Parkinson’s Disease-Associated Pathology. *Vaccines* 2023, *11*, 1820

**DOI:** 10.3390/vaccines13050512

**Published:** 2025-05-13

**Authors:** Jun Sung Park, Riaz Ahmad, Kyonghwan Choe, Min Hwa Kang, Tae Ju Park, Myeong Ok Kim

**Affiliations:** 1 Division of Life Sciences and Applied Life Science (BK 21 Four), College of Natural Science, Gyeongsang National University, Jinju 52828, Republic of Korea; jsp@gnu.ac.kr (J.S.P.); riazk0499@gnu.ac.kr (R.A.); or k.choe@maastrichtuniversity.nl (K.C.); kmh1020@gnu.ac.kr (M.H.K.); 2 Department of Psychiatry and Neuropsychology, School for Mental Health and Neuroscience (MHeNs), Maastricht University, 6229 ER Maastricht, The Netherlands; 3 Haemato-Oncology/Systems Medicine Group, Paul O’Gorman Leukaemia Research Centre, Institute of Cancer Sciences, College of Medical, Veterinary & Life Sciences (MVLS), University of Glasgow, Glasgow G12 0ZD, UK; t.park.1@research.gla.ac.uk; 4 Alz-Dementia Korea Co., Jinju 52828, Republic of Korea

The authors would like to make the following corrections to this published paper [1].

In the original publication, there was an error in **Figure 4H**. The images in the SNpc OVA and KLH DAPI merged panels were arranged incorrectly. They have now been properly arranged in the correct order, as shown in the revised figure.

In the original publication, the images of the striatum region for two groups (CTL with N and OVA) were mistakenly shuffled in **Figure 5F**. The incorrect images have been removed, and the correct ones have been incorporated into the revised figure. 

The striatum Veh and OVA panels overlapped by mistake in **Figure 6E**. The incorrect image has been removed, and the correct one has been incorporated into the revised figure.

To prevent any misunderstandings and ensure clarity, the authors would like to add the following sentences into the Supplementary Material for Figures S3 and S4:

Figures S3 and S4 present images of the same samples from the same experimental groups, with distinct regional focuses, as follows:

- Figure S3 illustrates the substantia nigra pars compacta (SNpc) region exclusively.
- Figure S4 encompasses both the SNpc and the ventral tegmental area (VTA), resulting in an apparent overlap between the two figures.

This distinction highlights the broader anatomical representation in Figure S4 while focusing on the SNpc in Figure S3 for a targeted analysis.

Due to the large number of images, the panels for Striatum Veh and KLH and Striatum N and SNpc KLH were inadvertently shuffled in **Supplemental Figure S6**. The incorrect images have been removed, and the correct ones have been incorporated.

**Figure S6:**
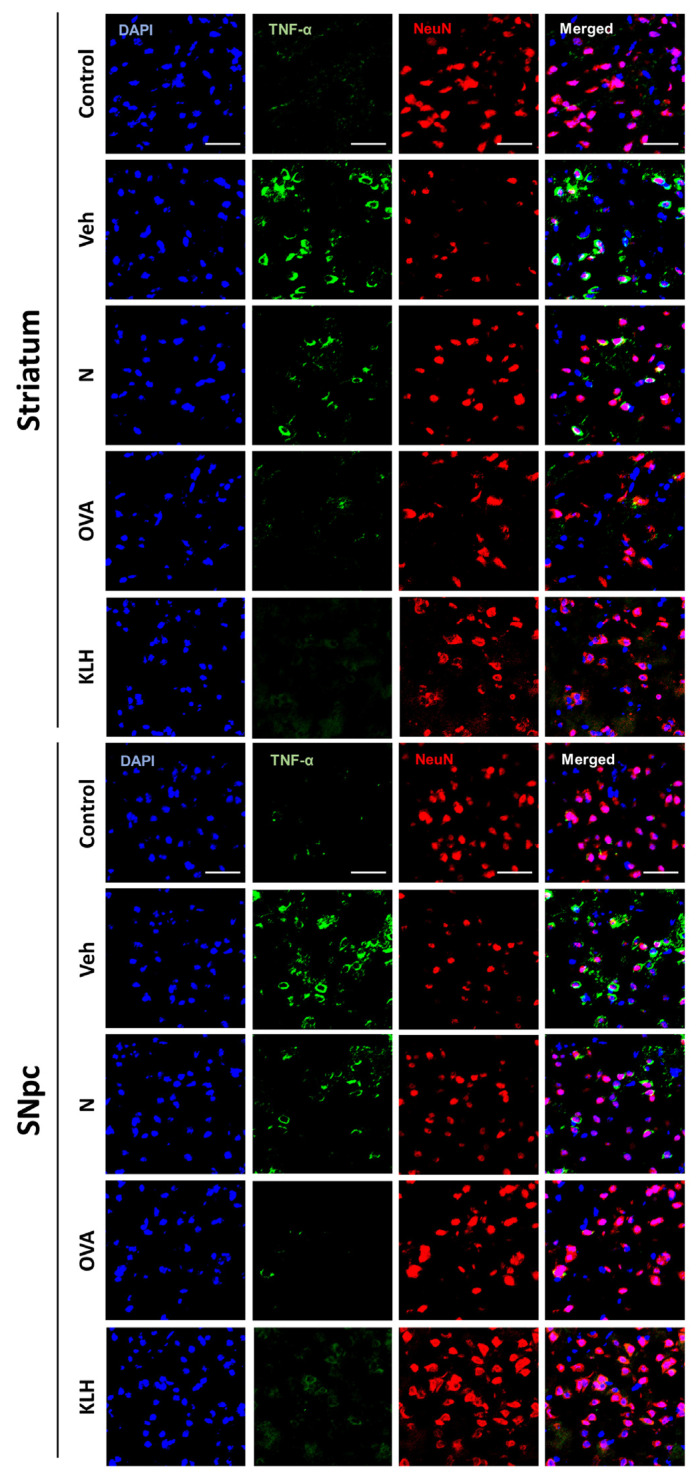
Immunofluorescence double staining of tumor necrosis factor alpha (TNFα) and neuronal nuclei (NeuN) in the striatum and substantia nigra pars compacta (SNpc). Blue represent DAPI, green represent TNF-α and red represent NeuN. Scale bar represent 20 μm.

The authors state that the scientific conclusions are unaffected. These corrections were approved by the Academic Editor. The original article has been updated.

## Figures and Tables

**Figure 4:**
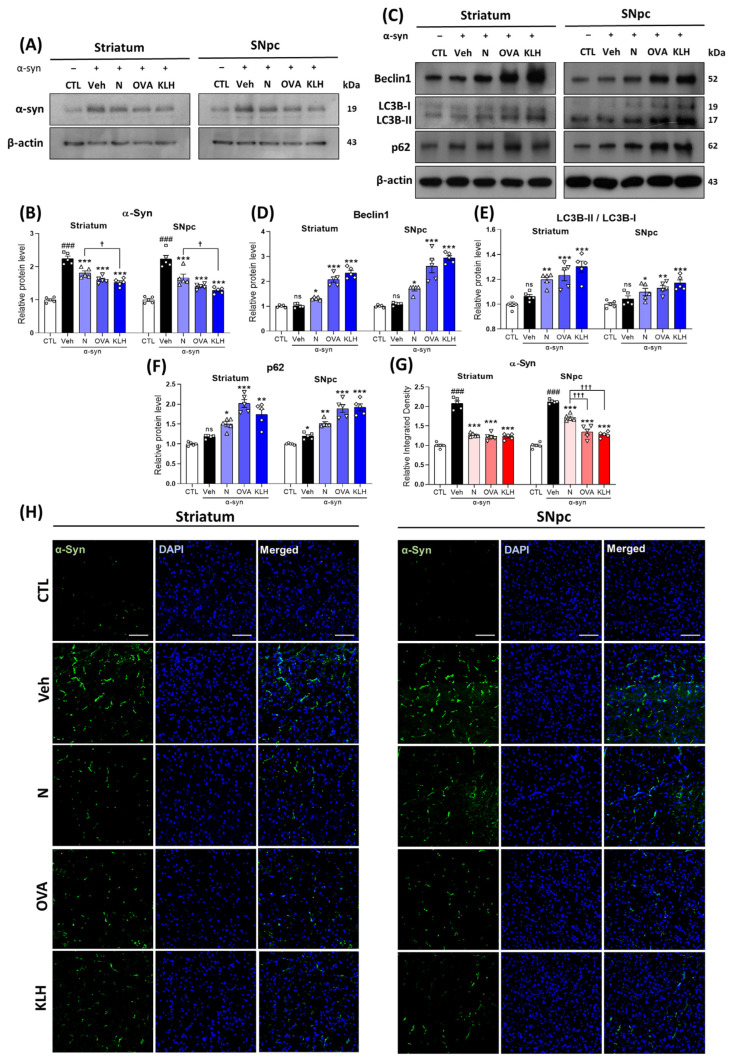
Epitope treatment reversed the α-syn and autophagy levels in the brain. (**A**,**B**) Western blot analysis showing the expression of α-syn in the striatum and SNpc (*n* = 5 per group). (**C**–**F**) Western blot analysis showing the expression of autophagy-related markers, i.e., (**D**) beclin-1, (**E**) LC3B-II/I, and (**F**) p62 in the striatum and SNpc (*n* = 5 per group). (**G**,**H**) Immunofluorescence staining measuring the expression of α-syn (green) co-stained with DAPI (blue) in the striatum and SNpc (*n* = 5 per group). Scale bar present 50 μm. Comparisons: ^#^ control (CTL) with saline-treated (Veh) α-syn-induced PD model; * Veh group with epitope treated group [non-carrier protein (N) and carrier-protein (OVA and KLH); ^†^ Non-carrier protein (N) with carrier protein (OVA and KLH). Data are presented as mean ± SEM. *^/†^ *p* < 0.05, ** *p* ≤ 0.01, ^###/^***^/†††^ *p* ≤ 0.001, and non-significant (ns).

**Figure 5:**
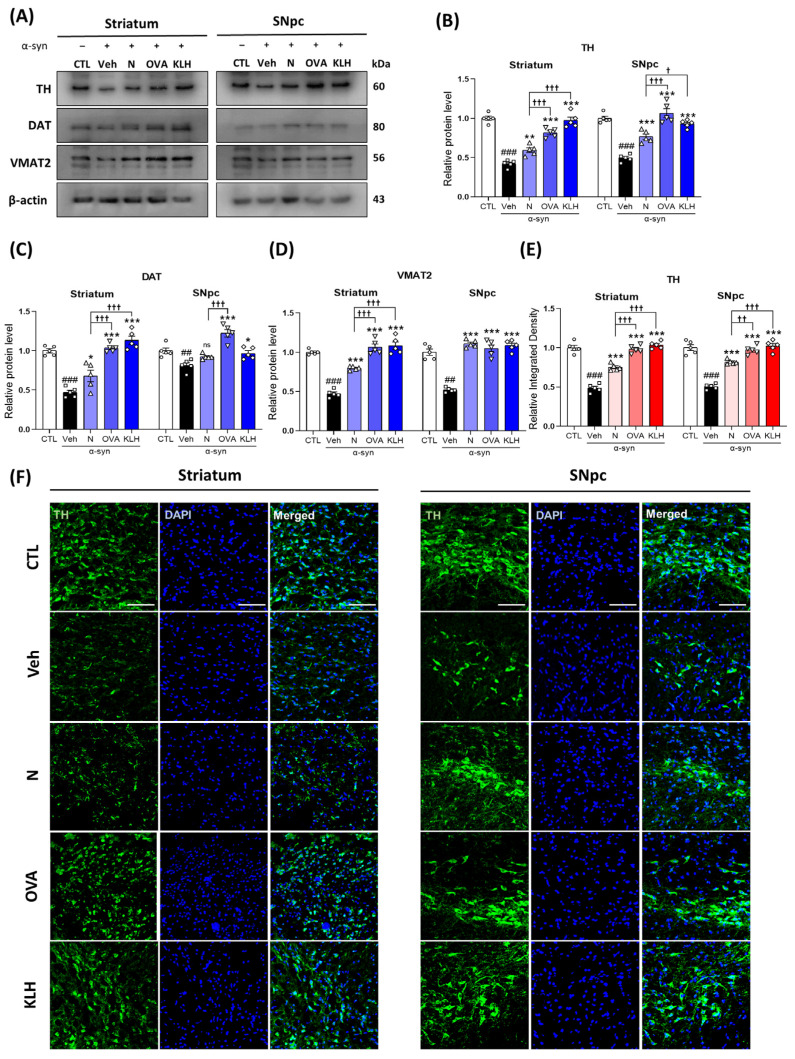
Epitope treatment increased dopamine-related markers in the brain. (**A**–**D**) Western blot analysis measuring the expressions of (**B**) tyrosine hydroxylase (TH), (**C**) dopamine transporter (DAT), and (**D**) vesicular monoamine transporter 2 (VMAT2) in the striatum and SNpc (*n* = 5 per group). (**E**,**F**) Immunofluorescence staining measuring the expression of TH (green) co-stained with DAPI (blue) in the striatum and SNpc (*n* = 5 per group). Scale bar present 50 μm. Comparisons: ^#^ control (CTL) with saline-treated (Veh) α-syn-induced PD model; * Veh group with epitope-treated group [non-carrier protein (N) and carrier-protein (OVA and KLH); ^†^ Non-carrier protein (N) with carrier protein (OVA and KLH). Data are presented as mean ± SEM. *^/†^ *p* < 0.05, **^/††^ *p* ≤ 0.01, ^###/^***^/†††^ *p* ≤ 0.001, and non-significant (ns).

**Figure 6:**
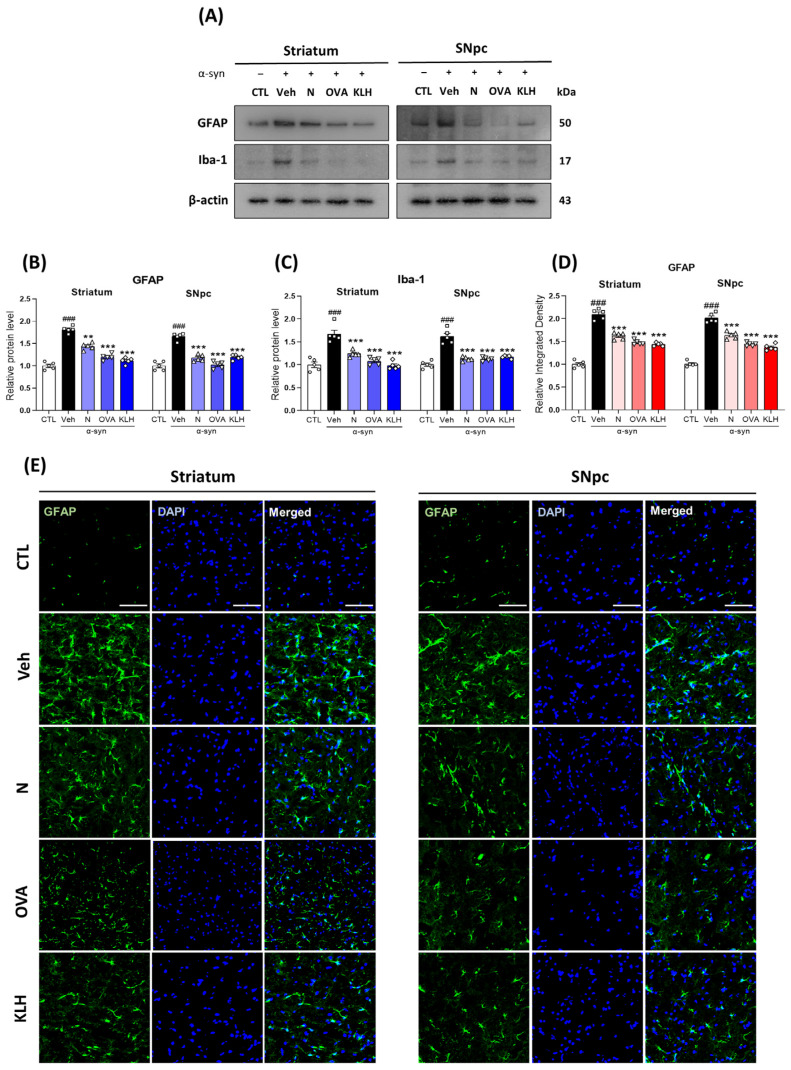
Epitope treatment reversed the glial cell activation in the brain. (**A**–**C**) Western blot analysis of (**B**) glial fibrillary acidic protein (GFAP) and (**C**) allograft inflammatory factor 1 (Iba-1) in the striatum and SNpc of mice (*n* = 5 per group); (**D**,**E**) Immunofluorescence staining measuring the expression of GFAP (green) co-stained with DAPI (blue) in the striatum and SNpc (*n* = 5 per group). Scale bar present 50 μm. Comparisons: ^#^ control (CTL) with saline-treated (Veh) α-syn-induced PD model; * Veh group with epitope-treated group [non-carrier protein (N) and carrier-protein (OVA and KLH). Data are presented as mean ± SEM. ** *p* ≤ 0.01, ^###/^*** *p* ≤ 0.001, and non-significant (ns).
