# Supplementary material for: Immunization Effects of a Novel α-Synuclein-Based Peptide Epitope Vaccine in Parkinson’s Disease-Associated Pathology
Source: Vaccines (Basel). 2023 Dec 5;11(12):1820. doi: 10.3390/vaccines11121820 (PMC10748214; doi:10.3390/vaccines11121820)
Supplement: Supplementary file 1 [file vaccines-11-01820-s001.zip › vaccines-2665446-supplementary.pdf]

Supplementary Materials

Immunization Effects of a Novel α-Synuclein-Based Peptide Epitope Vaccine in Parkinson's Disease-Associated Pathology

Jun Sung Park [1,†], Riaz Ahmad [1,†], Kyonghwan Choe [1,2,†], Min Hwa Kang [1], Tae Ju Park [3] and Myeong Ok Kim [1,4,*]

[1] Division of Life Sciences and Applied Life Science (BK 21 Four), College of Natural Science, Gyeongsang National University, Jinju 52828, Republic of Korea; jsp@gnu.ac.kr (J.S.P.); riazk0499@gnu.ac.kr (R.A.); k.choe@gnu.ac.kr or k.choe@maastrichtuniversity.nl (K.C.); kmh1020@gnu.ac.kr (M.H.K.)
[2] Department of Psychiatry and Neuropsychology, School for Mental Health and Neuroscience (MHeNs), Maastricht University, 6229 ER Maastricht, The Netherlands
[3] Haemato-Oncology/Systems Medicine Group, Paul O'Gorman Leukaemia Research Centre, Institute of Cancer Sciences, College of Medical, Veterinary & Life Sciences (MVLS), University of Glasgow, Glasgow G12 0ZD, UK; t.park.1@research.gla.ac.uk
[4] Alz-Dementia Korea Co., Jinju 52828, Republic of Korea
* Correspondence: mokim@gnu.ac.kr; Tel.: +82-55-772-1345
† These authors contributed equally to this work.

Supplementary Figure

(A) Bepipred Linear Epitope Prediction 2.0 Results

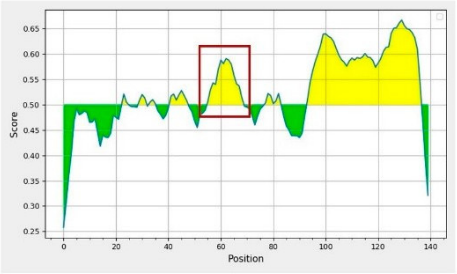

Predicted peptides :

No.	Start	End	Peptide	Legth
1	24	25	QG	2
2	30	32	AGK	3
3	34	36	KEG	3
4	42	48	SKTKEGV	7
**5**	**56**	**69**	**AEKTKEQVTNVGGA**	**14**
6	78	83	AQKTVE	6
7	94	137	FVKKDQLGKNEEGAPQEGILEDMPVDPDNEAYEMPSEEGYQDYE	44

(B) Bepipred Linear Epitope Prediction Results

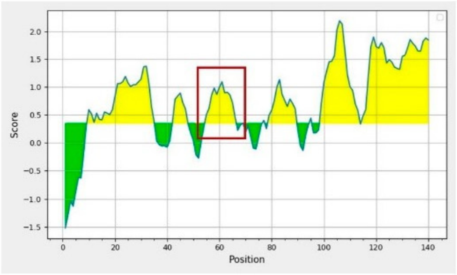

Predicted peptides :

No.	Start	End	Peptide	Legth
1	10	35	KAKEGVVAAAEKTKQGVAEAAGKTKE	26
2	42	47	SKTKEG	6
**3**	**55**	**66**	**VAEKTKEQVTNV**	**12**
4	69	69	A	1
5	77	77	V	1
6	79	89	QKTVEGAGSIA	11
7	95	95	V	1
8	99	113	QLGKNEEGAPQEGIL	15

(C) Emini Surface Accessibility Prediction Results

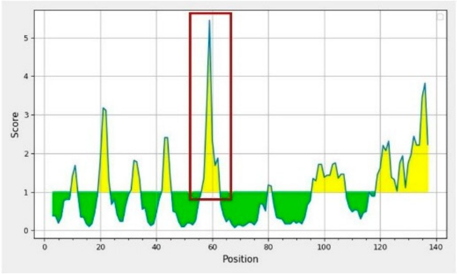

Predicted peptides :

No.	Start	End	Peptide	Legth
**1**	**57**	**62**	**EKTKEQ**	**6**
2	96	107	KKDQLGKNEEGA	12

Supplementary Figure S1. Algorithms for predicting B cell epitope of α-syn using the Immune Epitope Database (IDEB). **(A)** Bepipred Linear Epitope Prediction 2.0 and the predicted peptides. **(B)** Bepipred Linear Epitope Prediction and the predicted peptides. **(C)** Emini Surface Accessibility Prediction and the predicted peptides.

(A) **Epitope Candidate's Docking Score**

No.	**Epitope 1 (VAEKTKEQVT)**	Epitope 2 (AEKTKEQVTN)	Epitope 3 (EKTKEQVTNV)
1	**122.65**	106.89	96.84
2	**119.29**	105.21	96.46
3	**115.51**	100	93.5
4	**106.8**	99.63	88.53
5	**104.59**	95.97	88.37
6	**101.97**	95.97	87.25
7	**101.43**	95.4	83.59
8	**101.14**	91.63	83.05
9	**100.38**	91.53	82.76
10	**90.26**	90.46	81.13

(B) **Prediction of Toxic Peptides**

Peptide Seq	QM score	Prediction	Hydrophobicity	Hydropathicity	Hydrophilicity	Charge	Mol wt
VAEKTKEQVT	-10.9	**Non-Toxin**	-0.32	-0.95	0.79	0	1132.42

(C)

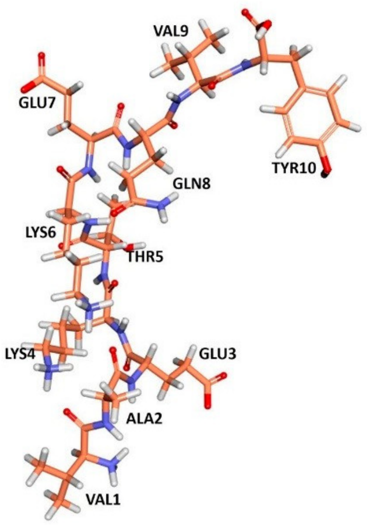

Epitope (V A E K T K E Q V T)

interaction	Chain	Interaction residues
Conventional Hydrogen Bond	A	TRP33
		ASP56
		HIS102
	B	TYR48
		ARG53
		SER55
		ASN94
	H	ALA158
		THR160

interaction	Chain	Interaction residues
Carbon Hydrogen bond	A	LYS31
		ALA32
		SER100
	B	PRO54
		SER55
	H	SER156
Pi-Donor Hydrogen Bond	B	PHE31
Pi-Sigma	B	TYR48
Pi-Pi T-shaped	B	PHE31
Alkyl	B	PRO90
Pi-Alkyl	B	PHE31
		HIS33
	H	ALA158

Supplementary Figure S2. Examination of the three candidate peptides. **(A)** Docking score via Genetic optimization for ligand docking (GOLD). **(B)** Selected epitope showing as non- toxic in the toxicity prediction model. **(C)** Structural conformation of the epitope and its interaction residues.

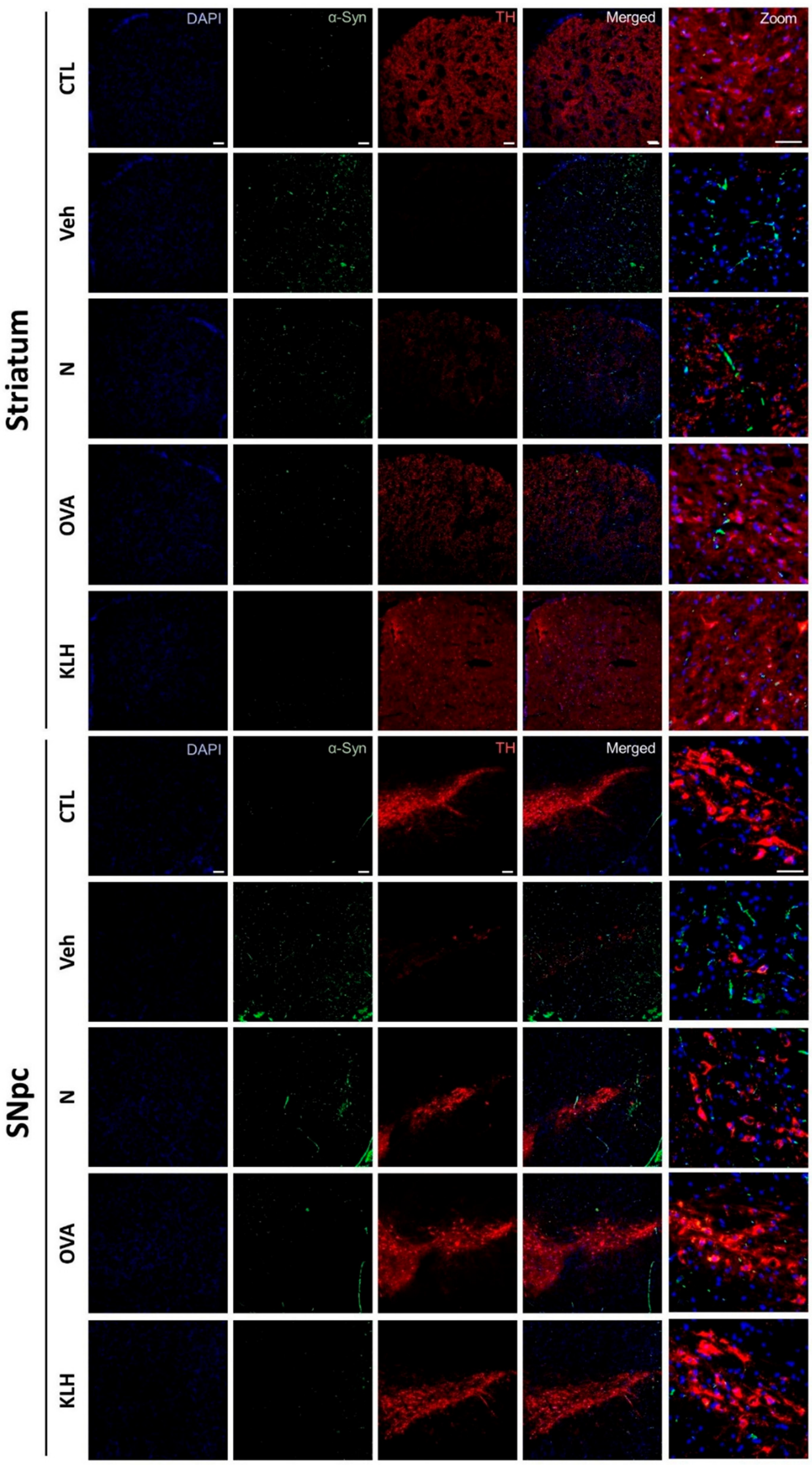

Supplementary Figure S3. Immunofluorescence double staining of α-synuclein (α-syn) and tyrosine hydroxylase (TH) in the striatum and the substantia nigra pars compacta (SNpc). Blue represent DAPI, green represent α-syn, and red represent TH. Scale bar represent 50μm and zoom is 20μm.

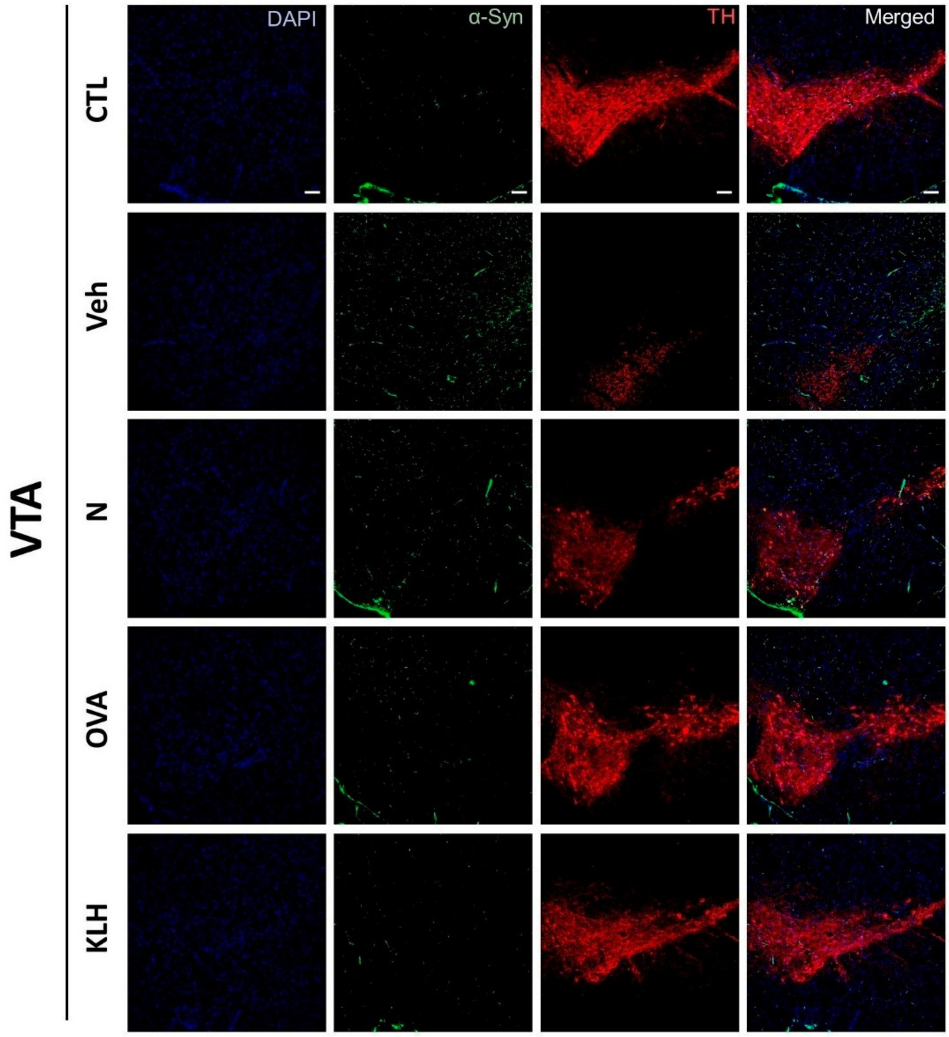

Supplementary Figure S4. Immunofluorescence double staining of α-synuclein (α-syn) and tyrosine hydroxylase (TH) in the ventral tegmental area (VTA). Blue represent DAPI, green represent α-syn, and red represent TH. Scale bar represent 50μm.

Figures S3 and S4 present images of the same samples from the same experimental groups, with distinct regional focuses, as follows:

- Figure S3 illustrates the substantia nigra pars compacta (SNpc) region exclusively.
- Figure S4 encompasses both the SNpc and the ventral tegmental area (VTA), resulting in an apparent overlap between the two figures.

This distinction highlights the broader anatomical representation in Figure S4 while focusing on the SNpc in Figure S3 for a targeted analysis.

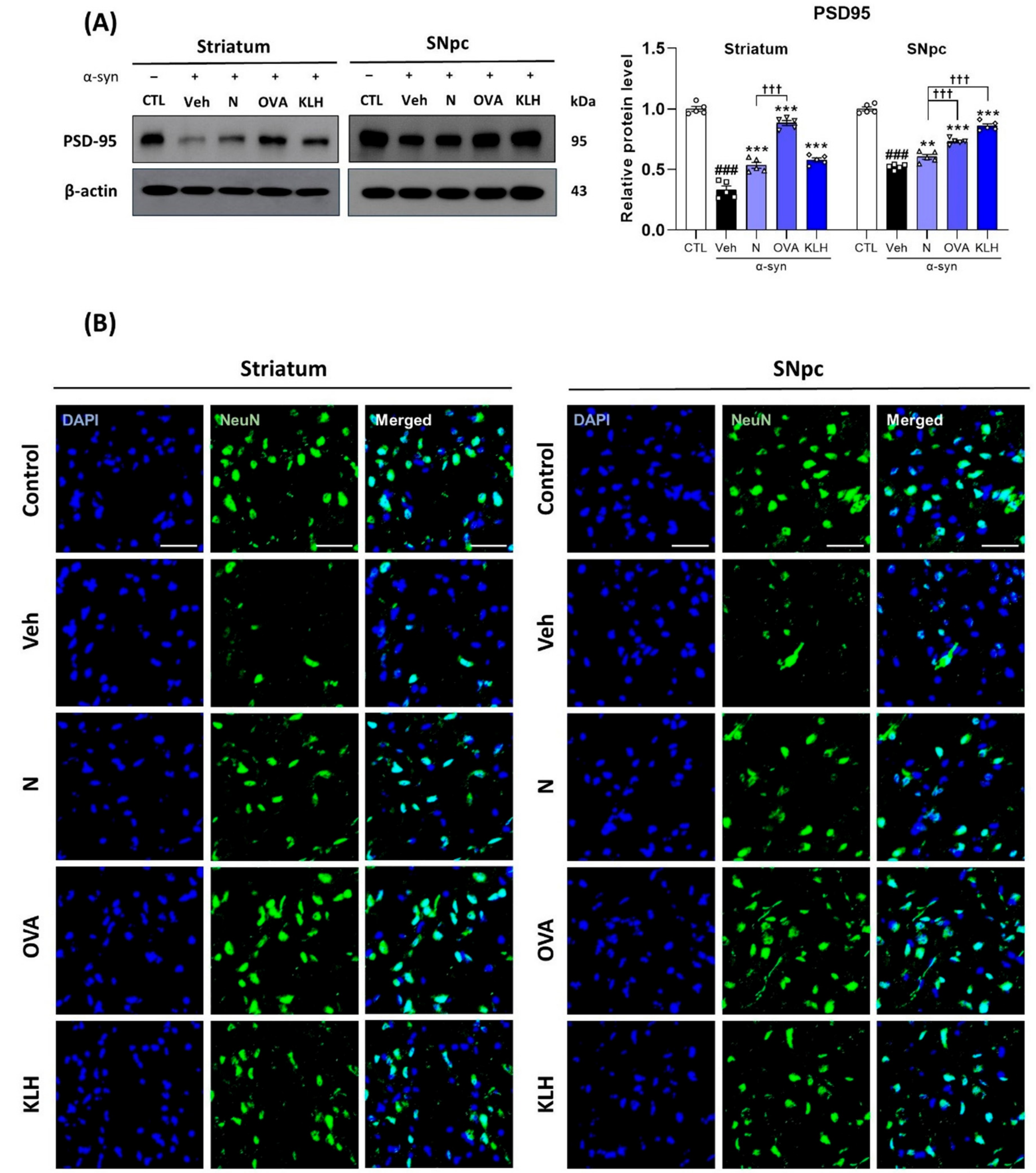

Supplementary Figure S5. Peptide-based epitope vaccines increased neurons in the striatum and substantia nigra pars compacta (SNpc). **(A)** Western blot analysis showing increased expression of postsynaptic density protein 95 (PSD-95) in the striatum and SNpc (n = 5 per group). **(B)** Immunofluorescence staining of neuronal nuclei (NeuN) stained with DAPI in the striatum and SNpc. Scale bar represent 20µm. Comparisons: #control (CTL) with saline-treated (Veh) α-syn-induced PD model; *Veh group with epitope treated group [non-carrier protein (N) and carrier-protein (OVA and KLH); †Non-carrier protein (N) with carrier protein (OVA and KLH). Data are presented as mean ± SEM. $^{\#\#/**/\dagger\dagger}$ $p \leq 0.01$ and $^{\#\#\#/***/\dagger\dagger\dagger}$ $p \leq 0.001$.

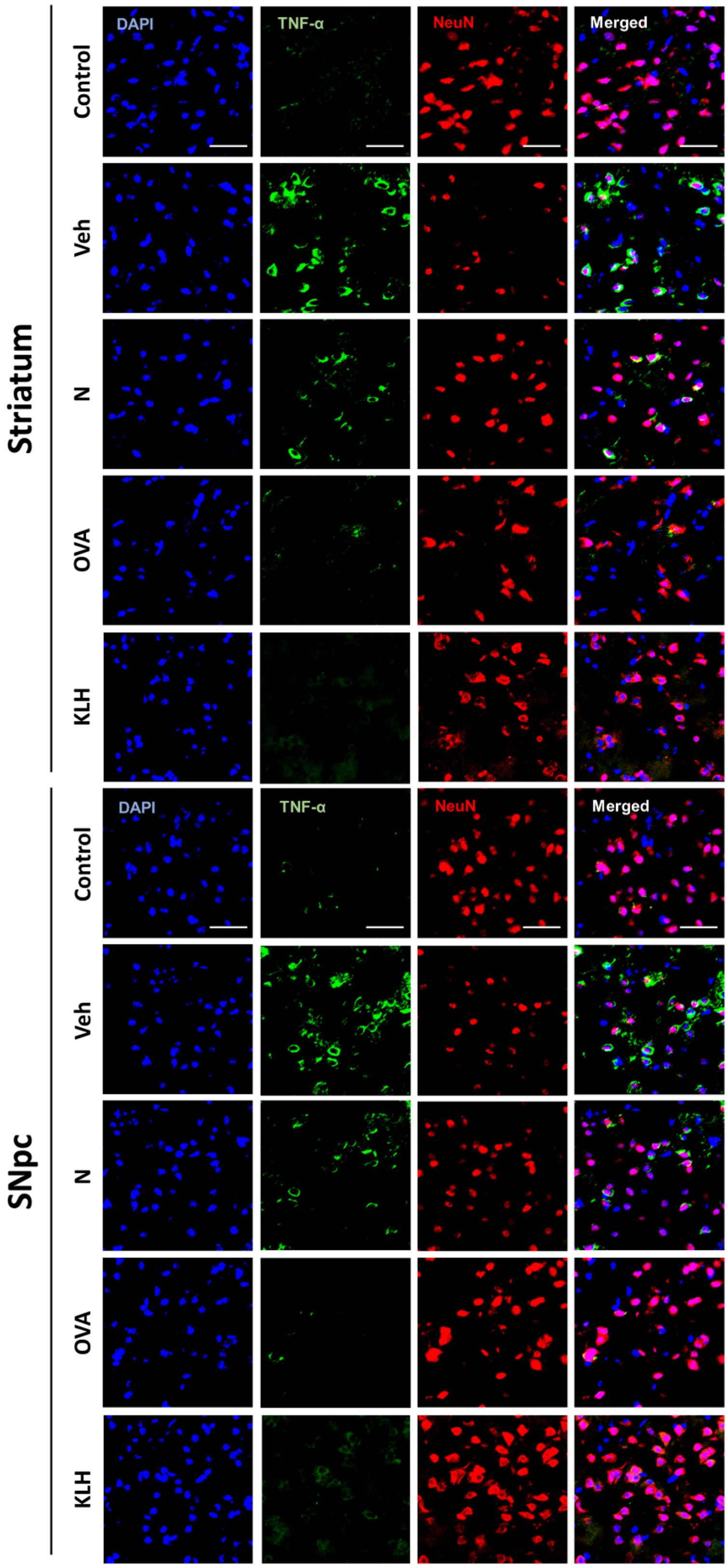

Supplementary Figure S6. Immunofluorescence double staining of tumor necrosis factor alpha (TNFα) and neuronal nuclei (NeuN) in the striatum and substantia nigra pars compacta (SNpc). Blue represent DAPI, green represent TNF-α and red represent NeuN. Scale bar represent 20μm.
